# Post‐transplant cyclophosphamide as sole GHVD prophylaxis after matched reduced‐intensity conditioning allotransplant

**DOI:** 10.1002/ctm2.1242

**Published:** 2023-04-26

**Authors:** Amandine Le Bourgeois, Maxime Jullien, Alice Garnier, Pierre Peterlin, Marie C. Béné, Thierry Guillaume, Patrice Chevallier

**Affiliations:** ^1^ Hematology Department Nantes University Hospital Nantes France; ^2^ INSERM UMR1232 CRCINA IRS‐UN University of Nantes Nantes France; ^3^ Hematology Biology Nantes University Hospital Nantes France

**Keywords:** allogeneic, ATG, clofarabine, GVHD prophylaxis, matched, PTCY

## Abstract

**Background:**

Post‐transplant cyclophosphamide (PTCY) alone as graft‐versus‐host disease (GVHD) prophylaxis may avoid/reduce short‐ and mid‐term toxicities of drugs commonly used for GVHD prophylaxis, accelerate immune reconstitution after the graft to decrease infections and facilitate the early integration of adjunct maintenance therapies to prevent relapse.

**Objective:**

A prospective phase 2 study was designed in order to assess the feasibility and safety of PTCY as a sole GVHD prophylaxis in adult patients receiving a Baltimore‐based reduced‐intensity conditioning (RIC) peripheral blood (PB) allogeneic hematopoietic stem cell transplantation (Allo‐HSCT) with a matched donor.

**Study design:**

Patients were planned to be included stepwise up to 59 evaluable PTCY recipients, in order to be able to stop the protocol in case of excessive corticosteroid resistant grade 3–4 severe acute GVHD (aGVHD). Because a high incidence of grade 2–4 aGVHD was observed after analysis of the first 27 patients, the protocol was amended to test the addition of 1 day of anti‐thymoglobulin to PTCY. In spite of this, the trial had to be stopped after 38 treated patients, because of an unacceptable rate of grade 3–4 aGVHD. Donors were matched related to 12 patients and unrelated to 26.

**Results:**

With a median follow‐up of 29.6 months, 2‐year overall, disease‐free and GVHD‐free relapse‐free (GRFS) survivals were respectively 65.4%, 62.1% and 46.9%. Cumulative incidences of grade 2–4 and 3–4 aGVHD at day 100 were 52.6% and 21.1%, respectively, while that of moderate/severe chronic(c) GVHD was 15.7% at 2 years. Addition of ATG to PTCY did influence neither aGVHD, cGVHD nor GRFS.

**Conclusion:**

Despite paradoxically good survivals, especially GRFS, this study failed to demonstrate that PTCY (± ATG) alone can be used for Baltimore‐based RIC PB Allo‐HSCT with matched donors. Other combinations should be tested to try and avoid long‐term use of immunosuppressive drugs following Allo‐HSCT in this setting.

## Graphical abstract

This was a prospective phase 2 study assessing the feasibility and safety of post‐transplant cyclophosphamide (PTCY) as a sole graft‐versus‐host disease (GVHD) prophylaxis in adult patients receiving a matched Baltimore‐based reduced‐intensity conditioning peripheral blood (PB) allogeneic hematopoietic stem cell transplantation (Allo‐HSCT). Despite paradoxically good survivals, this study failed to demonstrate that PTCY can be used alone in this setting because of an unacceptable high rate of grade III‐IV acute GVHD (21%). Other combinations should be tested to try and avoid long‐term prescription of immunosuppressive drugs following Allo‐HSCT in this setting.

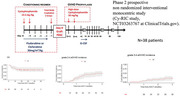

## 1 INTRODUCTION

In allogeneic hematopoietic stem‐cell transplantation (Allo‐HSCT), the use of high‐dose post‐transplant cyclophosphamide (PTCY) has revolutionized graft‐versus‐host disease (GVHD) prophylaxis, allowing the safe implementation of haploidentical Allo‐HSCT in recent years.^1^ As PTCY reduces significantly the incidence of both acute (aGVHD) and chronic (cGVHD), it has been thereafter considered in matched related/unrelated settings in combination with one or more immunosuppressive drugs, such as calcineurin inhibitors (ciclosporine [CsA], tacrolimus), mTOR inhibitors (sirolimus), inhibitors of DNA synthesis/replication (methotrexate [MTX], mycophenolate mofetil, [MMF]) or polyclonal anti‐T cells antibodies (anti‐human thymocyte immunoglobulins [ATG]).^2^ These therapies have also been compared to standard GVHD prophylaxis (especially CsA+MMF or CsA+MMF+ATG) showing mostly comparable survivals and reduced GVHD incidence.^3^, ^4^, ^5^, ^6^, ^7^, ^8^, ^9^ A prospective study comparing PTCY versus ATG in the matched setting should be published soon showing also no differences between both GVHD prophylaxis on outcomes.^10^

The ultimate step would be to administer PTCY alone in the aim to avoid/reduce short‐mid and/or long‐term toxicities (mainly infections and renal toxicity) of drugs commonly used for GVHD prophylaxis, accelerate immune reconstitution after the graft to decrease infections and facilitate the early integration of adjunct maintenance therapies to prevent relapse. The feasibility of such an approach has been validated in the context of myeloablative conditioning (MAC) allotransplant with both matched sibling (MSD) or unrelated (MUD) donors and bone marrow (BM) as source of graft. Indeed, from four studies in adults, incidences have been reported between 43% and 59% for grade 2−4 aGVHD, 10 and 15% for grade 3−4 aGVHD and 10% and 16% for cGVHD. Event‐free (EFS) and overall (OS) survivals were between 39% and 62% and between 55% and 67%, respectively in these studies.^11^, ^12^, ^13^, ^14^ Incidentally, it was also observed that grade 2 aGVHD was associated with significantly better OS and EFS, suggesting that it might be detrimental to not develop any GVHD.^14^ Nonetheless, survivals results were even more impressive in a cohort of 11 pediatric and young adult patients, showing a total absence of both aGVHD and cGVHD while EFS and OS were 42% and 54% at 2 years.^15^ Finally a recent Phase 3 trial showed that BM grafts followed by PTCY alone provided similar cGVHD (moderate or severe) or relapse‐free survival when compared to a same platform but with tacrolimus and methotrexate or CD34‐selected peripheral blood stem cell (PBSC) grafts.^16^

In the reduced‐toxicity conditioning (RTC)/reduced‐intensity conditioning (RIC) setting, one study for severe aplastic anemia patients showed also that PTCY alone was associated with low rates of aGVHD (grade 2−4: 22% and grade 3−4 11.1%) and cGVHD (22.7%) in patients undergoing sibling PBSC Allo‐HSCT.^17^ However, three other studies^18^, ^19^, ^20^ (with several limitations) showed disappointing results in terms of grade 3−4 aGVHD (22%, 27% and 80%) while grade 2−4 aGVHD incidences were quite comparable (58%, 45%, 80%) to those of the MAC BM grafts mentioned above. These high incidences may be related to the use of PBSC, the preferred source of HSCT for RTC/RIC, which are known to contain 10 fold more T‐cells in the graft compared to BM. Moreover, the majority of recipients were high‐risk older patients with advanced/active disease. Finally, it may also be hypothesized that the conditioning regimens (two fludarabine/busulfan RTCs and one combining fludarabine/carmustine/ melphalan) may have by themselves also influence GVHD occurrence, as reported before.^21^

Here, it was hypothesized that the use of Baltimore‐based RIC regimens with low‐dose total body irradiation,^22^, ^23^ a standard in the haplo‐HSCT setting, might allow to better control donor T‐cells and thus decrease grade 3−4 aGVHD incidence after PBSC matched Allo‐HSCT with PTCY as a sole GVHD prophylaxis.

## 2 METHODS AND PATIENTS

### 2.1 Study design and inclusion criteria

This trial was built as a prospective non‐randomized interventional monocentric phase 2 study (Cy‐RIC study, NCT03263767 at ClinicalTrials.gov). The main objective was to evaluate the feasibility and safety of using only PTCY, without CsA nor MMF, after a matched Allo‐HSCT. Adult patients (18–70 years old) with a good performances status (ECOG ≤2) receiving an RIC‐PBSC Allo‐HSCT with a 10/10 HLA MSD or unrelated (MUD) donor were eligible. Other inclusion criteria were: women with childbearing potential under efficient birth control method during the trial and up to 12 months after cyclophosphamide cessation, men under efficient birth control method during the trial and up to 6 months after cyclophosphamide cessation, negative serology to B and C hepatitis and HIV, written signed informed consent form and social security coverage. Exclusion criteria were: eligible to a MAC regimen, other progressive malignant disease or history of prior other malignancy in the last 2 years, with the exception of a curatively‐treated basal cell or cervix in situ carcinoma, progressive mental illness disease, pregnancy or breastfeeding, serious uncontrolled concomitant infection, contra‐indications to Allo‐HSCT (left ventricular ejection fraction < 45%, pulmonary impairment with <40% lung carbon monoxide diffusing capacity, creatinine clearance <60 mL/min, transaminases > 5x upper normal range [UPN] or bilirubin > 2 UPN), contra‐indications to cyclophosphamide (urinary tract infections, acute urothelial toxicity due to cytotoxic chemotherapy or radiotherapy, obstruction of urine flow, pre‐existing hemorrhagic cystitis, yellow fever vaccination), and finally cardiac condition preventing high dose cyclophosphamide utilization (New York Heart Association functional class II, III or IV, rhythmic, valvular or ischemic cardiomyopathy). Only patients having received the planned 2 days of PTCY were considered for analyses. If not, patients were replaced one by one. The study was approved by the Comité de Protection des Personnes Sud Est 1 de Saint‐Etienne (France), and the Agence nationale de sécurité du médicament. All patients provided informed consent.

### 2.2 Conditioning and GVHD prophylaxis

**FIGURE 1:**
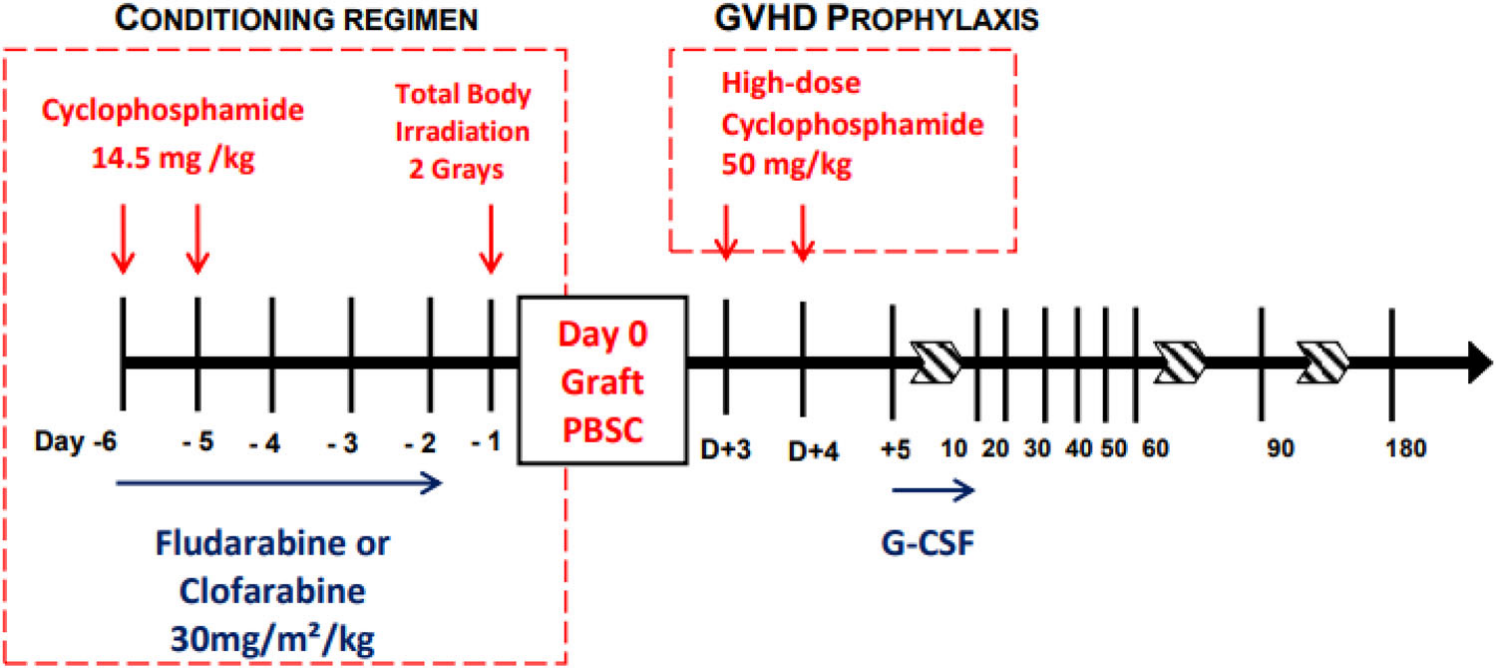
Trial backbone.

The Baltimore platform with 2 days of PTCY 50 mg/kg/day(D), D+3 and D+4 was used for conditioning regimen in all patients, with either fludarabine for lymphoid disease^22^ or clofarabine for myeloid disease.^23^ (Figure 1)

### 2.3 Primary objective and GVHD assessment

The primary objective was the incidence of corticosteroid‐resistant (CR) grade 3−4 aGVHD within the first 100‐days post‐transplant. Acute and cGVHD were graded according to Mount Sinai International Consortium^24^ and National Cancer Institute (NCI) criteria,^25^ respectively. Cortico‐resistance was defined initially as GVHD progression after 5 to 7 days of treatment with methylprednisolone (MP) 2 mg/kg per day equivalent or lack of improvement after 14 days of treatment with MP 2 mg/kg per day equivalent. More stringent criteria proposed by Greinix et al.,^26^ then Mohty et al.,^27^ were considered after October 2020.

According to statistical rules,^28^ 59 evaluable patients (having effectively received PTCY) had to be included. We had to include successively 3, 3, 6, 15, 15 and 17 patients waiting day 100 of the last patient per group to consider involving the next group.

The trial had to be interrupted in case of documentation of ≥2 CR 3−4 aGVHD among the first 3 patients, ≥3 CR 3−4 GVHD among the first 6 patients, ≥4 CR 3−4 GVHD among the first 12 patients, ≥6 CR 3−4 GVHD among the first 27 patients, ≥8 CR 3−4 GVHD among the first 42 patients and finally as soon as ≥9 CR 3−4 GVHD for the last patients to be included. This stepwise strategy would allow to halt the trial early enough in case of an excessive rate of deleterious severe aGVHD.

However, as described below, because a high incidence of grade 2−4 aGVHD was observed after analysis of the first 27 patients (but not the criteria for stopping the trial), an amendment was accepted in November 2020 to test the addition of 1 day of ATG 2.5 mg/kg day‐2 to PTCY for the next 32 patients to be included. ATG was chosen instead of CsA to avoid mid‐term immunosuppressive treatment.

### 2.4 Hematopoietic reconstitution, engraftment, and chimerism

Time to neutrophil recovery was defined as the first of 3 consecutive days in which the absolute neutrophil count exceeded .5 × 10^9^/L, and platelet recovery was defined as the first of 3 days with a count of 50 × 10^9^/L without a need for platelet transfusion. Primary graft failure was defined as persistence of aplasia after day +30 without relapse or by documentation of autologous reconstitution after transplant. Secondary graft failure was defined as reoccurrence of aplasia or autologous reconstitution after documented successful engraftment. Allogeneic or autologous reconstitutions were evaluated by quantitative polymerase chain reaction (Q‐PCR) evaluation of differential short tandem repeat DNA sequences as previously described.^29^ Mixed and full donor chimerism were defined by the presence of 5−94% and at least 95% of donor T cells, respectively, while engraftment was defined as sustained neutrophil recovery with full or mixed donor chimerism documentation.

### 2.5 Supportive care

Anti‐pneumocystis prophylaxis consisted of administration of trimethoprim‐sulfamethaxole from the end of aplasia until CD4+ lymphocyte recovery >200/mm^3^. Antiviral therapy consisting in administration of valacyclovir (1 g per day) for prevention of herpes simplex virus (HSV) and varicella zoster (VZV) infections was done in all patients for at least 1 year after transplant. Letermovir (480 mg per day), was used also in patients with a cytomegalovirus (CMV) positive serology from October 2019. Foscavir, gancyclovir or valgancyclovir were used pre‐emptively in patients showing 2 successive CMV viral load > 3 log/mL or in patients with documented CMV disease. Rituximab 375 mg/m^2^/week 4 weeks was used pre‐emptively in patients showing two successive Epstein Barr virus (EBV) positive PCR > 4 log/mL or in patients with refractory chronic GVHD or EBV‐associated lymphoma. CMV, EBV, adenovirus and HHV6 PCR were performed twice a week the first 2 months then once a week up to day 100 post‐transplant.

We used also prophylactic fluconazole (400 mg per day until day 100) in all patients. Patients with documented grade 2−4 acute GVHD were switched to posaconazole instead of fluconazole until the end of the corticosteroids therapy. Prophylactic use of immunoglobulins was done in patients with repeated infections during follow‐up. All of the patients received granulocyte colony‐stimulating factor (G‐CSF) during aplasia following transplant.

### 2.6 Immune reconstitution

Monocytes, T, B, and natural killer (NK) lymphocytes recovery in the blood was assessed for each engrafted patient with no relapse and no death before day day100 at various period of time: before transplant, at 3, 6, 9 and 12 months after allo‐HSCT. Absolute numbers of monocytes and total lymphocytes were collected. The CD3+ T cells, the CD4+ and CD8+ T cell subsets, the CD19+ B cells and the CD56+ NK lymphocytes were quantified using standard flow cytometry. Absolute numbers of cells were calculated by multiplying the peripheral white blood cell count by the percentage of positive cells.

### 2.7 Statistical analysis

Only patients having received the planned 2 days of PTCY were considered for analyses. If not, patients were replaced one by one.

Recorded clinical outcomes after transplantation included time to neutrophil and platelet engraftment, graft failure, time of onset and severity of aGVHD, cGVHD, relapse incidence (RI), non‐relapse mortality (NRM), as well as GVHD‐free/relapse‐free (GRFS), disease‐free (DFS) and overall (OS) survivals. OS was defined as the time from day 0 of allo‐HSCT to death or last follow‐up for survivors. DFS was defined as time from day 0 of allo‐HSCT to time without evidence of relapse or disease progression. Relapse was defined as any event related to re‐occurrence of the disease. NRM was defined as death from any cause without previous relapse or progression. GRFS was defined as being alive with no previous grade III‐IV aGVHD, no extensive cGVHD and no relapse.^30^, ^31^ Probabilities of OS and DFS were calculated using the log‐rank test and Kaplan–Meier graphical representation. Probabilities of RI, NRM and GVHD were calculated using the cumulative incidence (CI) procedure, and comparisons were performed using the Gray test. Analyses of severe toxicity were achieved by tabulation of the incidence of adverse events with NCI Common Terminology Criteria for Adverse Events, Version 5.0. Univariate and multivariate analyses were considered for acute and chronic GVHD and GRFS and OS. The variables considered for uni and multivariate analyses were : age (< vs. > = median), gender (male vs. female), type of disease (myeloid vs. lymphoid), disease‐risk index (DRI), (low/intermediate vs. high/very‐high risk), HCT‐CI (< 3 vs. > = 3), disease staus (complete remission vs. active disease), donor type (sibling vs. matched unrelated), ABO compatibility (compatible vs. other), donor/recipient CMV status (‐/‐ vs. other), CD34 stem cells infused (< vs. > = median), CD3 cells infused (< vs. > = median), type of GVHD prophylaxis (PTCY vs. PTCY+ATG) and aGVHD (grade 0–1 vs. grade 2 and grade 0–2 vs. grade 3−4).

All analyses were performed on August 2022 using the R statistical software version 3.2.3 (available online at http://www.R‐project.org). The univariate and multivariate analyses were conducted using Cox proportional hazards regression. When included as a covariate, GVHD was treated as a time‐dependent variable. Mantel‐Byar test and Simon‐Makuch plot were used to analyze survival data in relation to aGVHD. All tests were two‐sided and *p* values < .05 were considered as indicating a statistically significant association.

## 3 RESULTS

### 3.1 Patient characteristics

Between February 2018 and November 2021, a total of 47 adults were included, 38 receiving the 2 days of PTCY as required. The reasons for not receiving PTCY was cardiac contra‐indication (*n* = 4), relapse before conditioning initiation (*n* = 2), change of conditioning (*n* = 1), decision not to perform the graft (*n* = 1) and trial cessation (*n* = 1).

These 38 patients were considered for analyses since the criteria to stop the trial was reached after inclusion of the 38th patient (*n* = 8 patients/38 with corticosteroid resistant grade 3−4 aGVHD). There were 26 males and 12 females with a median age of 58 years old (yo) (range: 26−70). There were 31 patients with myeloid disease and 7 with lymphoid disease. Donors were 12 MSD and 26 MUD (Table 1).

**TABLE 1:** Patient characteristics.

Patients, *N* = 38	
**Gender**: male/female	26/12
**Median age**: years (range)	58 (26–70)
**Disease**: myeloid/lymphoid	31/7
AML/MDS/CMML/CML/MF/mixed syndrome	10/7/2/1/8/3
Ph+ALL/transformed CLL/prolymphocytic T/biphenotypic AL/NHL	1/1/1/1/3
**Status at transplant**	
CR1/CR2/active/PR2/PR3	14/4/18/1/1
**Previous autograft/allograft**	1/2
**Disease‐risk‐index**	
Low+intermediate/ high+very‐high	27 (1/26)/11 (10/1)
**Hematopoietic cell transplantation (HCT)‐specific comorbidity index** :0–2/ >2	19/19
**Conditioning regimen**	
Baltimore with fludarabine/with clofarabine	7/31
**GVHD prophylaxis**	
PTCY alone/PTCY+ 1 day of rabbit ATG	27/11
**Donors**	
Median age: years (range)	34.5 (19‐64)
Sibling (brother/sister)/Matched unrelated(male/female)	12 (8/4)/26 (19/7)
Female to male	6
**ABO compatibility**	
Compatible, minor incompatibility/major incompatibility	26/4/8
**CMV status**	
D‐/R‐	20
D+/R‐	5
D‐/R+	7
D+/R+	6
**Median CD34 stem cells graft infused: 10^6^/Kg of recipient**	7.54 (1.40–13.51)
**Median CD3+T cells graft infused: 10^7^/Kg of recipient**	18.20 (3–43)

Abbreviations: AL, acute leukemia; AML, acute myeloid leukemia; ATG, anti‐thymoglobuline; CLL, chronic lymphocytic leukemia; CML, chronic myeloid leukemia; CMML, chronic myelomonocytic leukemia; CR, complete remission; D, donor; MDS, myelodysplastic syndrome; MF, myelofibrosis; NHL, non‐Hodgkin lymphoma; Ph+ALL, Philadelphia chromosome positive acute lymphoblastic leukemia; PR, partial remission; PTCY, post‐transplant cyclophosphamide; R, recipient.

### 3.2 Hematopoietic reconstitution, engraftment, and chimerism

Two primary graft failures (5.2%) were documented, one in a 61 years old male patient with an active myelodysplasia at transplant who is still alive with active disease at 17 months post‐transplant, and one in a 62 years old male patient with active secondary myelofibrosis at transplant who is alive in remission at 18 months post‐transplant. Median days of neutrophil and platelet recoveries were 17 (range: 12−67) and 30 (range: 12−393) days, respectively. No secondary graft failure was observed. At day 30, 28 and 8 patients were documented with full and mixed donor chimerism, respectively. At day 100, considering alive patients in remission, all (*n* = 27) were documented with full chimerism.

### 3.3 Survivals

**FIGURE 2:**
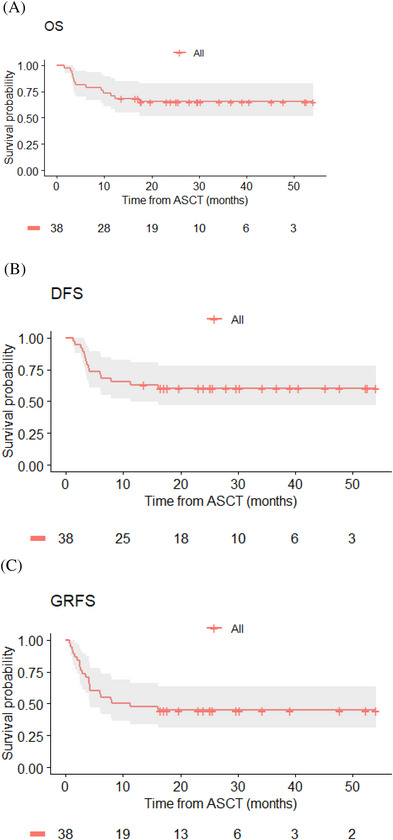
OS (A), DFS (B) and GRFS (C) for the whole cohort.

**FIGURE 3:**
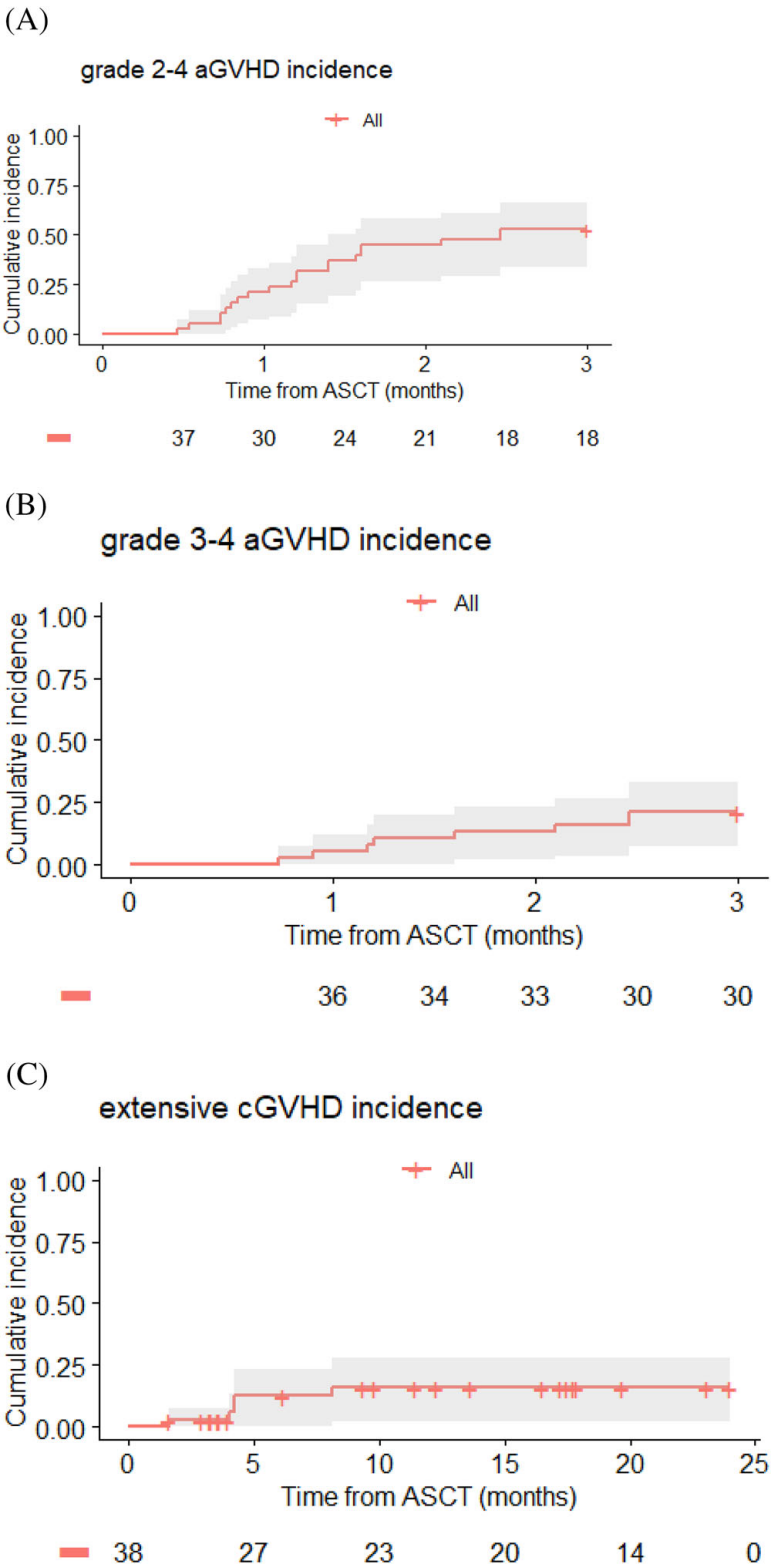
Cumulative incidences of grade 2−4 (A) and 3−4 (B) acute GVHD, and moderate/severe chronic GVHD (C).

With a median follow‐up of 29.6 months (range: 13.6–54) for alive patients (*n* = 25) at the time of analysis, 2‐year OS, DFS and GRFS were 65.4% (95% CI: 51−82), 62.1% (95% CI: 48−79) and 46.9% (95% CI: 33−66), respectively, all curves displaying a plateau after 17 months (Figures 2A,B and 3B). Thirteen deaths occurred, due to relapse (*n* = 4), aGVHD (*n* = 4; including 2 after donor lymphocyte infusion [DLI] for mixed chimerism), infections (non‐documented sepsis *n* = 2; COVID‐19 infection *n* = 1; adenovirus *n* = 1, aspergillosis and pseudomonas aeruginosa pneumonia *n* = 1). A maintenance therapy was not programmed in this study after transplant. However, three and one patients respectively received azacitidine + DLI and midostaurine as maintenance therapy post‐Allo‐HSCT.

### 3.4 Acute and chronic GVHD

**FIGURE 4:**
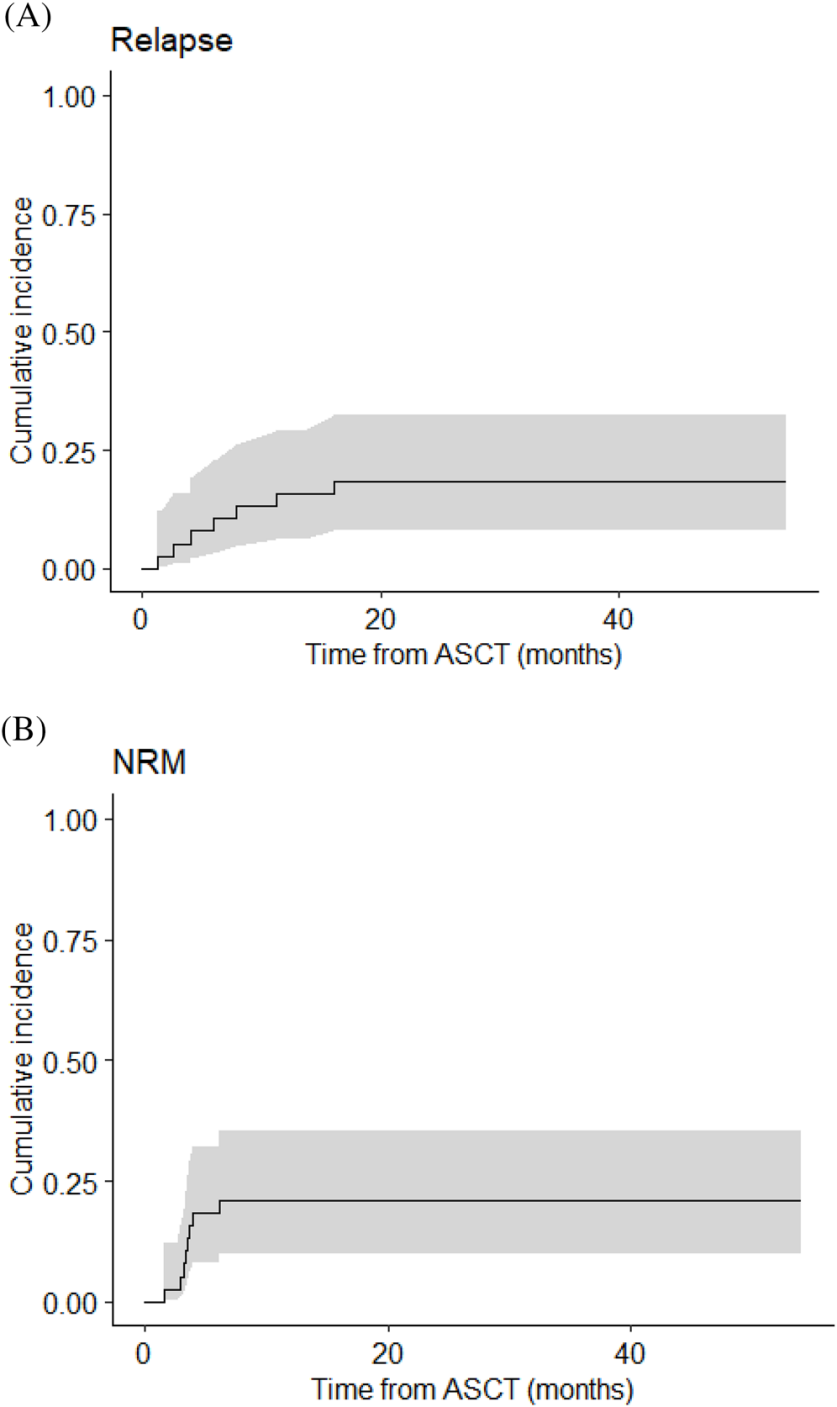
Cumulative incidences of relapse (A) and non‐relapse mortality (B).

Grade 2, 3 and 4 aGVHD occurred in 12 (cutaneous *n* = 8, gut *n* = 3, cutaneous+gut+liver *n* = 1), 2 (all gut) and 6 (gut *n* = 5, lung *n* = 1) patients, respectively. Corticosteroid resistance was observed in all eight patients with grade 3−4 aGVHD. CI of grade 2−4 (Figure 3A) and 3−4 aGVHD (Figure 3B) at day 100 were 52.6% (95% CI: 33.8%–66.1%) and 21.1% (95% CI: 7.0%–33.0%), respectively. Considering the eight patients with grade 3−4 corticosteroid resistant GVHD, the second line of treatment consisted of CsA and third line of MMF and/or jakavi and/or low dose methotrexate. At last follow‐up, two patients were alive without GVHD, while six died, two of GVHD and four of infections with a controlled GVHD.

Moderate/severe cGVHD occurred in 5/22 (22.7%) evaluable patients, including four still on immunosuppressive therapy at respectively 40, 28, 25 and 16 months post‐transplant. The CI of moderate/severe chronic GVHD was 15.7% (95% CI: 2.0%‐27.6%) at 2 years (Figure 3C).

### 3.5 Relapse and NRM

The CI of relapse was 18.5% (95% CI: 8.0%–32.5%) at 2 years (Figure 4A). Five patients relapsed (13%) and four died of relapse. One patient reached CR2 after salvage treatment and received a second Allo‐HSCT. She is still alive under preventive treatment of relapse by 5‐azacytidine + DLI. The CI of NRM was 21.1% (95% CI: 9.8%–32.5%) at 2 years (Figure 4B).

### 3.6 Severe toxicity

Some grade 3−4 severe adverse events were recorded: atrial fibrillation (*n* = 1), myocarditis (*n* = 1), pericarditis (*n* = 1), duodenal ulcer (*n* = 1), cholestasis (*n* = 8), diabetes mellitus (*n* = 1), depression (*n* = 1), hemoptysis (*n* = 1), pleurisy (*n* = 1), acute respiratory distress syndrome (*n* = 1).

### 3.7 Infections

Regarding viral infections, three EBV and three CMV reactivations, one herpetic gingivostomatitis, one adenovirus infection, one COVID‐19 infection and one BK virus hemorrhagic cystitis occurred. Regarding fungal infections, there were three aspergillosis and one *Candida kefyr* candidemia. As for bacterial infections, two *Pseudomonas aeruginosa* pneumoniae, one *Bacteroides fragilis* lung abscess, one nocardiosis, one *Enterococcus faecalis* septicemia and one *Enterobacter cloacae* septicemia were diagnosed. No parasitic infection was documented.

### 3.8 Immune reconstitution

Very interestingly, median monocyte counts remain in the normal range up to 1‐year post‐transplant while median B cells and NK cells counts were achieved as soon as 6 months post‐transplant. Median counts of total lymphocytes, CD3+ T cells, CD4+ T cells and CD8+ T cells increased all along the first year post‐transplant (Table 2).

**TABLE 2:** Immune reconstitution after transplant in engrafted patients with no relapse and/or death at day 100. All results expressed per mm^3^ with range.

	Pre‐graft, *n* = 27	+3 Months, *n* = 27	+6 Months, *n* = 23	+9 Months, *n* = 16	+12 Months, *n* = 21
Monocytes (NR: 150−900)	450 (5–2350)	420 (140–1130)	480 (210–970)	415 (280–870)	480 (230–750)
Lymphocytes (NR: 1500−4000)	800 (200–2250)	550 (160–1830)	650 (130–4180)	1130 (470–3410)	1000 (220–3350)
CD3+T cells (NR: 900−1800)	632 (101–1788)	296 (23–1184)	384 (92–3432)	512.5 (143–2343)	594 (72–2117)
CD4+T cells (NR: 500−1200)	370 (51–949)	99 (0–299)	134 (43–486)	203 (78–583)	214 (22–613)
CD8+T cells (NR: 300−700)	167 (52–1281)	132 (0–940)	241 (0–3098)	298 (63–1944)	341 (52–1455)
B cells (NR: 100−400)	22 (0–463)	10 (0–405)	110 (0–513)	177.5 (0–578)	136.5 (0–618)
NK cells (NR: 100−400)	68 (0–283)	198 (51–442)	199 (66–866)	224 (61–488)	153 (59–407)

Abbreviation: NR, normal range/mm^3^.

### 3.9 Univariate and multivariate analyses

No factor predicted GRFS nor grade 2−4 or 3−4 (Supplemental file) aGVHD in this cohort, especially when comparing PTCY versus PTCY+ATG sub‐groups (HR: 2.18; 95% CI .90–5.31, *p* = .08; HR: .71; 95% CI: .26–1.96, *p* = .50; and HR: 1.43; 95% CI: .34–6.01, *p* = .62, respectively) or MSD versus MUD (HR: 1.51; 95% CI: .55–4.13, *p* = .423; HR: .84; 95% CI: .33–2.11, *p* = .711; and HR: .76; 95% CI: .18–3.18, *p* = .707). The type of donor has no influence also on OS, DFS, NRM, relapse, or extensive chronic GVHD.

**FIGURE 5:**
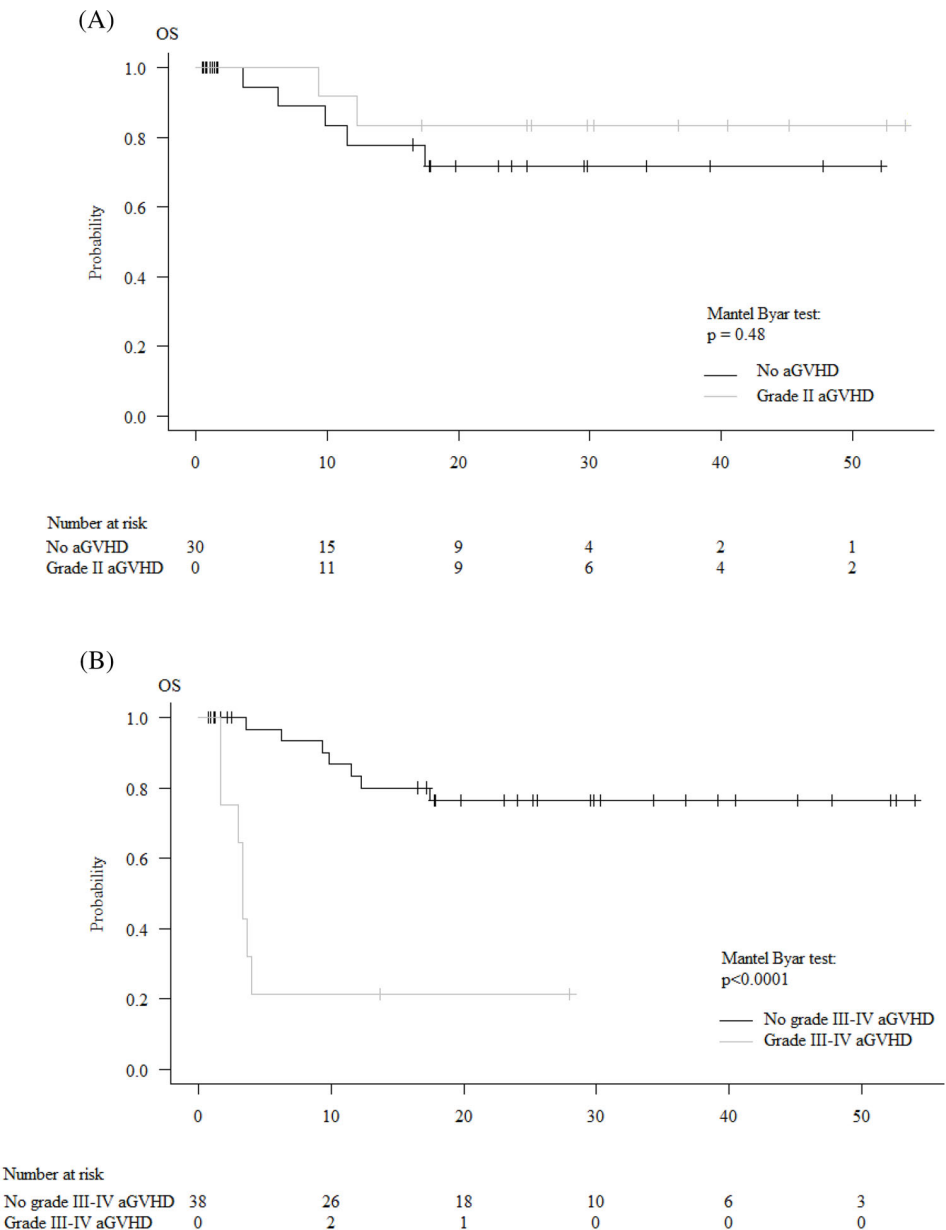
OS according to acute GVHD development: (A) comparison between grade 0−1 acute GVHD versus grade 2 acute GVHD patients; (B) comparison between grade 0−2 acute GVHD versus grade 3−4 acute GVHD patients.

Finally, a lower graft CD3+ T cells count was associated with lower moderate/severe cGVHD by univariate (HR: .82; 95% CI: .70–.96, *p* = .01) but not by multivariate analysis (HR: .94; 95% CI: .79–1.11, *p* = .46). The addition of ATG to PTCY influenced neither grade 2−4 aGVHD nor moderate/severe cGVHD. Except acute grade 3−4 GVHD (see below), an older age (>median) was the only factor associated with lower OS by univariate analysis (HR:1.07; 95% CI: 1.00–1.14, *p* = .049) and multivariate analysis (HR:1.07; 95% CI: 1.00–1.14, *p* = .064). Two‐year OS was not different between patients with grade 0–1 versus grade 2 aGVHD (71.8 ± 10% vs. 83.3 ± 10%, HR: .56; 95% CI: .11−2.88, *p* = .48) by univariate analysis. Grade 3−4 aGVHD (compared to grade 0−2) was significantly associated with lower OS by univariate (21.4 ± 14% vs. 76.4 ± 7%, HR: 8.14; 95% CI: 2.66−26.91, *p* = .0002) and multivariate (HR: 8.22; 95% CI: 2.36–28.64, *p* = .0009) analyses (Figure 5).

## 4 DISCUSSION

This phase 2 prospective study ultimately shows that the use of PTCY (+‐ATG) as a sole GVHD prophylaxis cannot be used safely in adults receiving a Baltimore‐based RIC regimen and PBSC as source of graft. Indeed, an unacceptable 21% incidence of grade 3−4 aGVHD (all corticosteroid resistant) was observed, leading to early discontinuation of the trial after induction of the 38th patients over the 59 initially planned. This incidence is similar to what has been reported by other studies, as described below, demonstrating clearly that patients receiving PBSC Allo‐HSCT after a RIC/RTC regimen are not good candidates to receive PTCY or PTCY/ATG as single GVHD prophylaxis.

With a Flu‐Bu3 RTC regimen and BM or PBSC as source of graft, Alousi et al. showed in a cohort of 49 mostly high‐risk patients, of whom 11 received ATG with PTCY, an incidence of 58%, 22% and 18% of grade 2−4, 3−4 aGVHD and cGVHD, respectively. DFS and OS were 26% and 33% at 2 years. When comparing this PTCY cohort to one receiving tacrolimus/methotrexate as GVHD prophylaxis, significant lower OS and higher rates of acute GVHD and NRM were observed with PTCY but the two cohorts were not well matched.^18^ Holtick et al., reporting on only 11 patients with lymphoma or myeloma receiving a PBSC Flu‐BU3 RTC regimen with PTCY alone, showed 45% and 27% of grade 2−4 and 3−4 aGVHD but only two mild cGVHD after relapse treatment, with 34% DFS and 64% OS at 2 years.^19^ A third study had to be stopped rapidly because four of five patients presented grade3‐4 aGVHD with PTCY alone after Allo‐HSCT with PBSC and a RIC regimen combining fludarabine/carmustine/melphalan.^20^

The hypothesis here was to suppress CsA (or Tacrolimus) and MMF not only to decrease toxicity and allow, at best, tolerable maintenance or targeted therapy after Allo‐HSCT, but also to decrease drastically the number of medications taken by the patients at home to improve their quality of life. It could allow also to shorten the time for immune reconstitution. The three‐drug prophylaxis (PTCY/CsA or tacrolimus/MMF) in PB matched Allo‐HSCT is generally associated with good survivals and low NRM and GVHD incidences.^32^, ^33^, ^34^ The combination of PTCY with two drugs is also supported by a retrospective study from the European Society for Blood and Marrow Transplantation (EBMT).^35^ However, short courses of bortezomib^36^, ^37^ or sirolimus^38^ with PTCY alone have been proven to provide similar results. This strengthened the concept of attempting to avoid short, mid or long‐term prescription of immunosuppressive drugs. And then, what could be the next step? Higher dose of ATG may be an alternative as various studies have shown reduced acute and/or chronic GVHD incidences combining PTCY+ATG 4.5 or 5 mg/Kg total dose in the HLA‐matched setting.^39^, ^40^, ^41^ Another possibility could be to combine PTCY with ATG and a short course of methotrexate (MTX, D1 D3 D6 +‐D11) as is done with calcineurin inhibitors in the MAC setting. Of note, the combination of clofarabine+ATG+short course of MTX has been already reported.^42^ Finally, another strategy might be to use BM instead of PBSC as stem cell source, but BM is rarely used in the context of RIC Allo‐HSCT.

Although it had to be halted, this trial nonetheless yielded relatively good survivals, especially GRFS. Very good survivals are suspected to be related to a strong graft versus leukemia effect associated with a high incidence of grade 2 aGVHD, as reported before.^14^ Of note, here, grade 2 aGVHD was not significantly associated with better survival compared to patients presenting with no or grade 1 aGVHD.

An elevated CD3+ graft content was associated with an increased incidence of all‐grade chronic GVHD (HR 1.36 [95% CI  =  1.06−1.74], *p*  =  .01) in the study by Mussetti et al.^43^ a study where patients received a haploidentical transplant with PTCY/MMF+‐CsA or tacrolimus or sirolimus, but this effect was confirmed only for the PBSC graft group. Conversely, the small effective of our study may explain that the CD3 graft content was not associated with chronic GVHD in multivariate analysis. However, it may be also explained by the fact that the median CD3+T cells graft infused in our cohort was lower (18.20 [3–43] 10^7^/Kg of recipient) compared to the study by Mussetti et al. (median of 23 [3–70]).^43^

Considering immune reconstitution, our study compares favorably for some cells subsets with the results of two studies using various conditionings and PTCY‐based prophylaxis in the setting of unrelated matched allotransplant. Comparing to Khimani et al.,^44^ median B and NK cell counts were higher at 3 and 6 months post‐transplant (but not CD4+ and CD8+ T cells) in our study. Comparing to Mehta et al.,^45^ median counts of total lymphocytes, CD3+ T cells, CD8+ T cells and NK cells were lower at 3 months compared to our study. This strengthens our hypothesis that a potential quicker immune recovery could be potentially achieved using PTCY in combination with short‐term other immunosuppressive treatment as GVHD prophylaxis in the setting of RIC PBSC matched Allo‐HSCT.

In conclusion, despite good survivals, PTCY alone does not appear to be immunosuppressive enough to allow an acceptable severe GVHD incidence after Baltimore‐based PB Allo‐HSCT. Thus, this specific approach must be avoided in the future. Other combinations will have to be tested in the future in order to combine tolerable post‐Allo‐HSCT maintenance and improved immune reconstitution and quality of life.

## CONFLICT OF INTEREST STATEMENT

The authors declare no conflict of interest.
